# LAMA2-Related Dystrophies: Clinical Phenotypes, Disease Biomarkers, and Clinical Trial Readiness

**DOI:** 10.3389/fnmol.2020.00123

**Published:** 2020-08-05

**Authors:** Anna Sarkozy, A. Reghan Foley, Alberto A. Zambon, Carsten G. Bönnemann, Francesco Muntoni

**Affiliations:** ^1^Dubowitz Neuromuscular Centre, Institute of Child Health, Great Ormond Street Hospital for Children, London, United Kingdom; ^2^Neuromuscular and Neurogenetic Disorders of Childhood Section, National Institute of Neurological Disorders and Stroke, National Institutes of Health, Bethesda, MD, United States; ^3^National Institute for Health Research Great Ormond Street Hospital Biomedical Research Centre, London, United Kingdom

**Keywords:** LAMA2, phenotype, biomarkers, clinical trial, natural history

## Abstract

Mutations in the *LAMA2* gene affect the production of the α2 subunit of laminin-211 (= merosin) and result in either partial or complete laminin-211 deficiency. Complete merosin deficiency is typically associated with a more severe congenital muscular dystrophy (CMD), clinically manifested by hypotonia and weakness at birth, the development of contractures of large joints, and progressive respiratory involvement. Muscle atrophy and severe weakness typically prevent independent ambulation. Partial merosin deficiency is mostly manifested by later onset limb-girdle weakness and joint contractures so that independent ambulation is typically achieved. Collectively, complete and partial merosin deficiency is referred to as LAMA2-related dystrophies (LAMA2-RDs) and represents one of the most common forms of congenital muscular dystrophies worldwide. LAMA2-RDs are classically characterized by both central and peripheral nervous system involvement with abnormal appearing white matter (WM) on brain MRI and dystrophic appearing muscle on muscle biopsy as well as creatine kinase (CK) levels commonly elevated to >1,000 IU/L. Next-generation sequencing (NGS) has greatly improved diagnostic abilities for LAMA2-RD, and the majority of patients with merosin deficiency carry recessive pathogenic variants in the *LAMA2* gene. The existence of multiple animal models for LAMA2-RDs has helped to advance our understanding of laminin-211 and has been instrumental in preclinical research progress and translation to clinical trials. The first clinical trial for the LAMA2-RDs was a phase 1 pharmacokinetic and safety study of the anti-apoptotic compound omigapil, based on preclinical studies performed in the *dy*^W^/*dy*^W^ and *dy*^2J^/*dy*^2J^ mouse models. This phase 1 study enabled the collection of pulmonary and motor outcome measures and also provided the opportunity for investigating exploratory outcome measures including muscle ultrasound, muscle MRI and serum, and urine biomarker collection. Natural history studies, including a five-year prospective natural history and comparative outcome measures study in patients with LAMA2-RD, have helped to better delineate the natural history and identify viable outcome measures. Plans for further clinical trials for LAMA2-RDs are presently in progress, highlighting the necessity of identifying adequate, disease-relevant biomarkers, capable of reflecting potential therapeutic changes, in addition to refining the clinical outcome measures and time-to-event trajectory analysis of affected patients.

## Introduction

The laminin α2–related muscular dystrophies (LAMA2-RDs) are a subtype of congenital muscular dystrophy (CMD) caused by recessive variants in the *LAMA2* gene [6q22–q23; OMIM*156225] (Helbling-Leclerc et al., 1995; Zhang et al., 1996). *LAMA2* encodes for the alpha-2 subunit of the heterotrimeric laminin-2 protein (made up of α2, β1, and γ1 subunits) with the α2 subunit called laminin-211 or merosin serving as a tissue-specific component of the extracellular matrix with a key role in myotubes stability and apoptosis (Vachon et al., 1996). The clinical spectrum of LAMA-RDs is wide, ranging from a severe, early-onset, and progressive presentation to a milder, later-onset form. To date, there are no effective treatments for LAMA2-RDs.

In this review article, we present a detailed overview of the most relevant clinical aspects of LAMA2-RDs and provide an update on translational developments, in particular natural history studies and available disease-related biomarkers.

## LAMA2-RD: Clinical Aspects

LAMA2-RDs are classically divided into two main phenotypic categories: a more common severe, early-onset form, presenting with features of CMD, also known as Merosin Deficient Congenital Muscular Dystrophy type 1A (MDC1A), and a much less common, milder, later-onset form often presenting with a phenotype suggestive of limb-girdle muscular dystrophy (LGMD) with prominent joint contractures. Severe LAMA2-RD is one of the most common forms of CMD, accounting for ~1/3 of patients with a diagnosis of CMD (Allamand and Guicheney, 2002; Muntoni and Voit, 2004; Sframeli et al., 2017), with an estimated prevalence in UK and Italy of 0.6–0.7/100,000 (Mostacciuolo et al., 1996; Norwood et al., 2009). The prevalence of the milder LAMA2-RD form is not fully known. The UK and Danish studies showed *LAMA2* variants in about 2–3% of patients with mild muscular dystrophies or LGMD (Løkken et al., 2015; Sframeli et al., 2017). As a general rule, patients with the severe CMD-like phenotype have a (virtually) complete absence of merosin as detected by immunohistochemical staining on muscle or skin biopsy. Conversely, patients with milder clinical presentations have some residual (or partial) merosin expression (Naom et al., 1998; Pegoraro et al., 1998; Topaloğlu et al., 1998; Tezak et al., 2003; Oliveira et al., 2008; Geranmayeh et al., 2010; Gavassini et al., 2011; Xiong et al., 2015). However, exceptions do exist and extreme intrafamilial variability is reported, suggesting that disease modifiers play a role in defining phenotypes and severity (Prandini et al., 2004; Geranmayeh et al., 2010).

### Severe LAMA2-RD

**Figure 1:**
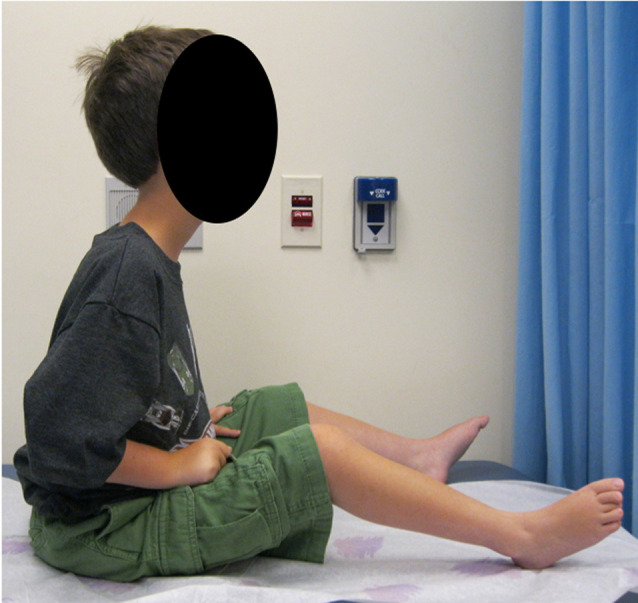
Clinical phenotype. A patient with complete merosin deficiency, with evidence of hip, knee, and ankle contractures with lordotic posture which is typical of LAMA2-RD patients from an early age (written informed consent for the publication of the clinical image was obtained from a parent of the patient).

The classic, severe LAMA2-RD presentation is a relatively homogenous CMD phenotype and is most typically associated with complete merosin deficiency on muscle biopsy immunohistochemical studies. Clinical hallmarks are early-onset severe hypotonia, axial weakness, inability to achieve independent ambulation, and elevated creatine kinase (CK) levels, commonly >1,000 IU/L (Figure 1). Progressive joint contractures, respiratory insufficiency, and scoliosis are observed in almost all patients. About 2/3 of patients are symptomatic at birth, with a further 1/3 with symptoms recognized by age 6 months of age (Geranmayeh et al., 2010; Xiong et al., 2015). Presenting symptoms include hypotonia, a weak cry, and reduced spontaneous movements. Respiratory problems, feeding difficulties, and mild distal contractures can also be present at birth, but severe arthrogryposis is not usually observed. At the onset, weakness primarily affects axial muscles, with severe head lag and predominant upper more than lower limb involvement. The great majority of patients present with motor developmental delay. Not infrequently, a sharp decline in motor function during the first weeks of life followed by a degree of improvement and partial attainment of motor milestones is subsequently observed during the first year/s of life (personal observation). While the majority of patients eventually attain trunk control, antigravity strength is typically not achieved in neck flexion, trunk flexion, and the deltoid muscles. Some patients achieve the ability to stand with support for variable periods; however, independent ambulation is only achieved exceptionally. A review of 33 LAMA2-RD patients documented independent ambulation in two patients with complete merosin deficiency, namely at 3.6 and 4 years of age (Geranmayeh et al., 2010). It is noteworthy that none of these two children had feeding or respiratory complications at the time of ambulation. A more recent review of a cohort of LAMA2-RD patients seen at the Dubowitz Neuromuscular Centre identified 6/42 patients (14%) who were ambulant with variable support and variable lengths of time (Zambon AA, Muntoni F and Sarkozy A, personal observation). Similar prevalences emerge from further published cohorts, overall indicating that ambulation during childhood (mostly with support and for limited periods only) is possible in up to about 10% of LAMA2-RD patients with complete merosin deficiency (Jones et al., 2001). Of note, the presence of residual merosin expression was demonstrated in biopsies of a number of these patients, and thus it is possible that in some of these patients with a relatively milder phenotype a low level of merosin expression provided a partial benefit.

Facial weakness, typically with drooling and elongated face, macroglossia, and the protruding tongue is common in patients with severe LAMA2-RD. A progressive limitation of extraocular movements, in particular of upward gaze, is noted as early as at 2 years of age, with clear ophthalmoparesis in the horizontal and upwards direction becoming more evident by the end of the first decade. Interestingly, a deficit of downwards movements or intrinsic muscles as well as ptosis are not observed (Philpot and Muntoni, 1999).

Progressive, restrictive pulmonary insufficiency due to weakness of intercostal and accessory muscles is the most common cause of morbidity and mortality in LAMA2-RD. Inefficient cough is usually present from the first months of life. Patients will often need the use of cough assistance during respiratory infections, with ~1/3 of patients requiring non-invasive ventilatory (NIV) support in early childhood. Patients started on NIV in the first year of life can sometimes be subsequently weaned-off (Geranmayeh et al., 2010). In a previously published Dubowitz Neuromuscular Centre series of patients, NIV was needed in 4/18 patients <5 years and 8/9 patients aged >10 years with complete merosin deficiency (Geranmayeh et al., 2010). The median age at NIV initiation reported in this cohort was approximately 13 years. Of note, while diaphragm excursion is well-preserved, on dynamic MRI imaging LAMA2-RD patients demonstrate reduced chest wall expansion (Foley AR and Bönnemann CG, personal observation). While invasive ventilation might become necessary for short periods and typically during times of respiratory infections, long-term use of invasive ventilation or use of ventilation via a tracheostomy is rare in pediatric LAMA2-RD patients.

Feeding is variably impaired in its oral, pharyngeal, laryngeal, and/or esophageal phases (Philpot et al., 1999a). Swallowing difficulties can lead to aspiration, recurrent chest infections, and failure to thrive. Macroglossia and facial weakness might further contribute to the defective oral phase in LAMA2-RD patients. Prolonged mealtimes can be distress for families and children. While gastrostomy tube placement is a common and effective procedure for weight and infections’ control, safe oral feeding has been observed post-gastrostomy insertion in ~60% of severe LAMA2-RD patients in a Dubowitz Neuromuscular Centre cohort.

Severe, progressive proximal and distal joints’ contractures, in upper and lower limbs, can be present from as early as birth (Prandini et al., 2004), with a considerable detrimental effect on motor function. Progressive scoliosis is common after 6 years of age, leading to a surgical correction in most patients. Severe lordosis, spine, and neck rigidity is also observed. Geranmayeh et al. (2010) reported scoliosis in 14/33 patients with complete merosin efficiency. Interestingly, only two of these 14 patients achieved some form of ambulation, and all but one had decreased or insufficient respiratory function, suggesting a positive correlation between the severity of motor, respiratory, and spinal involvement.

In pediatric patients, independent from the overall clinical severity, cardiac involvement is not often significant (Muntoni, 2003). However, several reports highlight the considerable frequency of subclinical cardiac involvement, in particular, right bundle branch block and left ventricular dysfunction (Spyrou et al., 1998; Finsterer et al., 2010), with rare reports of heart failure at various ages, and thus regular cardiac monitoring with cardiac rhythm assessment by Holter monitoring and cardiac imaging by echocardiogram is recommended in all LAMA2-RD patients.

**Figure 2:**
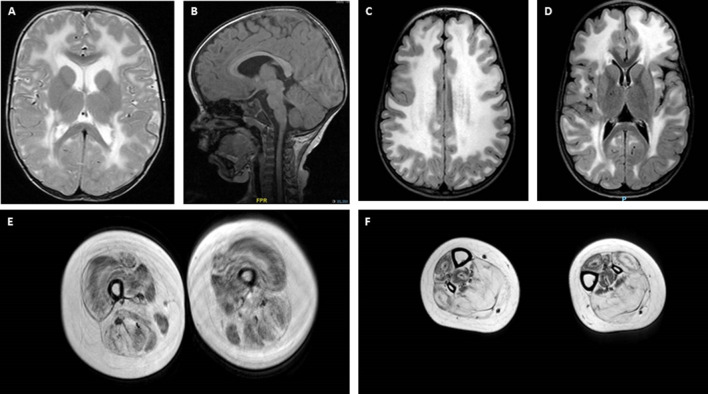
Brain and muscle imaging findings in LAMA2-RD. **(A–D)** Typical brain MRI findings in LAMA2-RD with abnormal appearance of myelin with sparing of the U fibers seen on T2 axial images **(A,C,D)**. T1 sagittal image **(B)** demonstrates evidence of occipital polymicrogyria, which has been observed in some LAMA2-RD patients and may predispose to occipital lobe seizures. Muscle MRI of the upper leg **(E)** and lower leg **(F)** in a 9-year-old patient with complete merosin deficiency with evidence of abnormal signaling in most muscles with relative sparing of the sartorius and gracilis muscles **(E)**. In the lower leg, the muscles of the anterior compartment seem relatively spared compared to muscles of the posterior compartment, namely the soleus and gastrocnemius muscles, which demonstrate the apparent replacement of muscle with fibrofatty tissue.

Central nervous system (CNS) and peripheral nervous system involvement is frequent in LAMA2-RD. Characteristic brain white matter (WM) hypointensity on T1 magnetic resonance imaging (MRI), and increased T2 signal in the periventricular and subcortical WM, are invariably observed in most patients older than 6 months (Farina et al., 1998; Philpot et al., 1999b; Leite et al., 2005), independently from clinically evident CNS involvement (Figures 2A–D). Cerebral atrophy and neuronal migration defects (such as focal cortical dysplasia, polymicrogyria, or cortical anomalies typically affecting the occipital regions) can be observed in ~10% to ~40% of patients from various series. Seizures, usually responsive to antiepileptic medications in the absence of an underlying cortical anomaly, are observed in up to ~30% of patients (Jones et al., 2001; Bönnemann et al., 2014). Seizures can be simple or complex partial episodes, occasionally spreading to secondary generalized tonic-clonic seizures (Prandini et al., 2004). The most consistent hypothesis regarding the etiology of these seizures is abnormal neuronal firing due to deficient neuronal migration in specific areas of the cortex (Ahmed et al., 2019), which may be present even when not appreciated on neuroimaging. Mild-to-moderate cognitive disability is reported in a small proportion of LAMA2-RD patients (Messina et al., 2010) and one series was associated with additional structural occipital cortex abnormalities (Mercuri et al., 1999). Mild, sensorimotor demyelinating neuropathy is commonly observed, but its contribution to muscle weakness is considered to be minimal in the human, while it plays a substantial role in mouse models of lama2 deficiency (Shorer et al., 1995; Previtali and Zambon, 2020).

Long term survival has not been well documented in LAMA2-RD. A life expectancy up to the third decade is usually observed, but death as early as the first decade has been described in some patients mainly due to complications of respiratory insufficiency (Philpot et al., 1995; Xiong et al., 2015).

### Mild LAMA2-RD

Patients with partial merosin deficiency present with a variable, milder clinical form of LAMA2-RD. Phenotypes range from CMD-like to milder and later presentations including late-onset LGMD-like phenotypes (Naom et al., 1998; Pegoraro et al., 1998; Topaloğlu et al., 1998; Di Blasi et al., 2000; Tezak et al., 2003; Gavassini et al., 2011; Løkken et al., 2015; Nelson et al., 2015; Harris et al., 2017). Partial LAMA2-RD with LGMD presentation is now also classified as LGMDR23 (Straub et al., 2018). The majority of patients with mild LAMA2-RD present their first symptoms during childhood, often with delayed motor milestones and raised CK values. However, a significant proportion of patients achieve and maintain independent ambulation. For example, one large series reported that >60% of patients with residual merosin gained independent or supported ambulation for variable lengths of time (Geranmayeh et al., 2010).

In partial merosin deficiency scoliosis is generally less frequent and progressive than in patients with complete merosin deficiency. Indeed, in a series with 13 patients with partial merosin deficiency, one had scoliosis (Geranmayeh et al., 2010). This patient, aged 15 years at the last assessment, was ambulant with support and presented severe contractures. Conversely, all but one of the 13 patients with partial merosin deficiency (who was aged 1.3 years at last assessment) showed variable degrees of joint contractures, suggesting mild LAMA2-RD has relevant contractual features. In keeping with this finding, recent reports provided evidence that residual merosin deficiency can also clinically manifest as a prominent contractual presentation resembling Emery Dreifuss MD (Nelson et al., 2015).

Patients with partial merosin deficiency are statistically less likely to require ventilatory support or enteral feeding during their lifetime with NIV needed in 1/13 patients with partial merosin deficiency vs. 13/33 patients with complete merosin deficiency (*P*-value 0.0354; Geranmayeh et al., 2010). Similarly, only one of seven patients with partial deficiency aged less than 5 years needed enteral feeding vs. 9/19 of patients with complete merosin deficiency.

Cardiac monitoring in patients with partial merosin deficiency has frequently detected subclinical, primary dilated cardiomyopathy as well as rhythm and conduction disturbances that could be potentially life-threatening, in particular after the third decade of life (Carboni et al., 2011; Marques et al., 2014; Nelson et al., 2015; Harris et al., 2017).

CNS involvement, with epilepsy, typical WM changes, and cortical alterations are not uncommon, and peripheral sensorimotor demyelinating neuropathy is also observed (Geranmayeh et al., 2010; Chan et al., 2014; Harris et al., 2017; Kim et al., 2017; Kubota et al., 2018).

### Genotype-Phenotype Correlations

Prediction of clinical severity is based not only on knowledge of the residual amount of merosin but also on location and mutational mechanism of the *LAMA2* variants (Ge et al., 2018; Oliveira et al., 2018). As of December 2017, the *LAMA2* gene variant database^1^ listed 309 disease-associated variants (de Oliveira et al., 2014). Overall, homozygous or biallelic loss-of-function mutations (including larger deletions/duplications) in *LAMA2* preferentially lead to severe phenotypes and complete merosin deficiency on muscle biopsy. Conversely, missense variants, in particular those occurring in the N-terminal region with preserved C-terminal expression, are associated with residual merosin expression and milder clinical presentations (Naom et al., 1998; Oliveira et al., 2008, 2014, 2018; Ding et al., 2016). Among these, the *LAMA2* variant c.2461A>C (p.Thr821Pro; in homozygosity or compound heterozygosity with loss-of-function variants) has now been described in several patients with milder EDMD or LGMD-like clinical presentations (Marques et al., 2014; Nelson et al., 2015), as well as in patients with mild, atypical forms of LAMA2-RD with predominant CNS involvement (Marques et al., 2014; Nelson et al., 2015; Oliveira et al., 2018). Conversely, in-frame deletions involving the G-domain can still result in a severe LAMA2-RD presentation, often with residual merosin on muscle biopsy, highlighting the importance of using antibodies directed towards different epitopes of the protein for the immunodiagnosis. A nonsense variant c.4645C>T; p.(Arg1549Ter) has now been associated with milder phenotype both in homozygosity and compound heterozygosity, possibly due to alternative in-frame splicing of exon 32 (Di Blasi et al., 2000, 2001; Geranmayeh et al., 2010). However, we identified the same truncating variant in patients with severe LAMA2-RD, suggesting that genotype-phenotype correlations are still challenging (personal observation). Intrafamilial clinical variability is often reported, one of the most extreme examples being an Italian sibship with two sisters carrying a homozygous loss-of-function *LAMA2* variant, presenting with severe and mild LAMA2-RD, respectively (Prandini et al., 2004).

### Diagnosis

The identification of two pathogenic variants in the *LAMA2* gene is the diagnostic gold standard for LAMA2-RD. However, diagnosis can be strongly aided by a combination of clinical features and results of investigations. Elevated serum CK (commonly >1,000 IU/L) is nearly a constant finding in all LAMA2-RD patients, though normal CK has been rarely reported (Sframeli et al., 2017). Brain, and increasingly also body, MRI may provide supportive diagnostic evidence in atypical patients and may support the pathogenicity of unclear *LAMA2* variants. For complete merosin deficiency patients, the role of muscle MRI may be less helpful than brain MRI, however, since muscles can demonstrate abnormal signaling on MRI from an early age (Figures 2E,F). Interestingly, muscle MRI features in the lower limbs of LAMA2-RD patients with milder phenotypes are similar to what has been reported in patients with COL6-related dystrophies, with the typical inside-out pattern of fatty replacement (Nelson et al., 2015; Harris et al., 2017).

**Figure 3:**
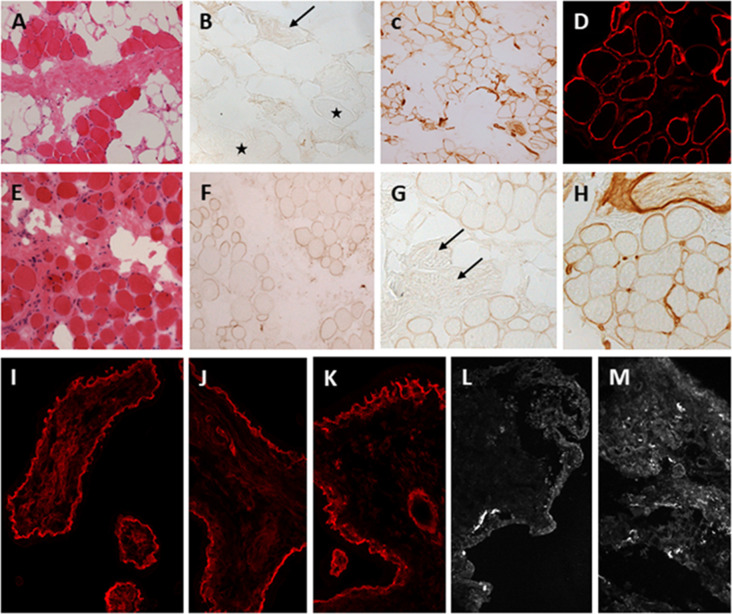
Laminin-211 deficiency in diagnostic quadriceps muscle biopsies and chorionic villus biopsies. Complete laminin-211 deficiency in quadriceps muscle biopsy **(A–D)**. Hematoxylin and eosin **(H & E)** stained section **(A)** shows marked dystrophic changes. Immunolabeling with the 300 kDa laminin-211 antibody **(B)** shows complete absence at the myofiber basal lamina **(B**, stars) and on an intramuscular motor nerve (**B**, arrow). There is widespread secondary upregulation of laminin alpha 5 **(C)**, whereas labeling with the IIH6 antibody against the glycosylated alpha-dystroglycan epitope appears normal. Partial laminin-211 deficiency in quadriceps muscle biopsy **(E–H)**. H&E stained section **(E)** shows moderate dystrophic changes. Immunolabeling with the 300 kDa laminin-211 antibody shows moderate, patchy reduction at the myofiber basal lamina **(F)** and complete absence on an intramuscular motor nerve (**G**, arrows). There is widespread secondary upregulation of laminin alpha 5 **(H)**. Prenatal testing for laminin-211 deficiency in chorionic villus biopsies **(I–M)**. Labeling of a positive control sample (fetus unaffected by LAMA2-RD) **(I)** with the 300 kDa laminin-211 antibody shows distinct membranous labeling at the trophoblastic basement membrane of chorionic villi. Example of a test case showing normal labeling **(J,K)** comparable to the positive control, and another case showing complete absence of laminin-211 at the trophoblastic basal lamina **(L,M)** of first-trimester chorionic villi. In this case, the complete absence of laminin-211 expression suggests that the fetus is affected by LAMA2-RD. Normal labeling for laminin-211 in chorionic villi does not exclude carrier status for LAMA2-RD.

Muscle biopsies of LAMA2-RD patients show dystrophic changes that can range from mild to severe (Figures 3A,E). Deficiency of laminin-211 can be demonstrated in sections using specific antibodies such as Alexis 4H8 recognizing the N terminal 300 kDa fragment of the protein. The deficiency ranges from partial in milder cases (Figures 3F,G) to complete in severe cases (Figure 3B) and is also observed in the intramuscular motor nerves (Figure 3B stars, 3G, arrows). Absence of laminin alpha 2 at the epidermal and adnexal basement membranes and intradermal sensory nerves can be demonstrated in skin biopsies, particularly in cases of complete laminin alpha 2 deficiency (Sewry et al., 1996, 1997). Thus, a skin biopsy can be offered as a less invasive alternative diagnostic tool in cases where a muscle biopsy is not feasible.

Next-generation sequencing (NGS) is now able to identify pathogenic *LAMA2* variants in close to 100% of patients with suspected LAMA2-RD (Oliveira et al., 2018). Of note, a considerable number of patients have been shown to carry variable-sized deletions/duplications in the *LAMA2* gene and thus, NGS with copy number variation testing is recommended in case two pathogenic variants are not found on NGS alone (Ge et al., 2018). Furthermore, deep intronic variants, synonymous variants leading to abnormal splicing, or copy neutral variations (such as inversions) can be identified by whole genome or RNA sequencing in patients that remain undiagnosed after whole gene sequencing (Gonorazky et al., 2019). Molecular preimplantation and prenatal diagnosis could be offered to families where two clear pathogenic changes are found in the previously affected child. As laminin-211 is expressed in trophoblasts from the 9th week of gestation, analysis of laminin-211 on fetal cells could be offered for families were two pathogenic variants were not identified (Vainzof et al., 2005; Figures 3I–M). However, interpretation of partial deficiencies is challenging and thus this analysis is only recommended in cases where complete merosin deficiency was observed in the index patient. At present, neonatal screening is not available for LAMA2-RD.

### Management

Clinical management of LAMA2-RD patients is focused on the prevention and treatment of complications. Early interventions with physical, occupational, and speech therapy should be arranged to optimize motor and cognitive development. Regular physical activity, including stretching of joints and providing orthosis for upper and lower limbs should be started from the time that a diagnosis of LAMA2-RD is suspected. Ongoing scoliosis monitoring and surgical intervention as needed should be offered to patients. Referral to respiratory care teams should be prompt, given the known natural history of respiratory insufficiency which can manifest with recurrent chest infections from the very first years of life in patients with complete merosin deficiency. Chest physiotherapy and the use of the Cough Assist machine can help in reducing hospitalizations and avoid potentially life-threatening episodes. Pulmonary surveillance includes pulmonary function tests, pulse oximetry, and sleep studies. For patients with complete merosin deficiency, the use of nocturnal NIV becomes necessary during the first decade of life. Weight monitoring from early infancy should focus on addressing feeding difficulties to minimize failure to thrive. As silent aspiration is possible in complete merosin deficiency patients, baseline swallowing evaluation should be obtained regardless of symptoms. Many patients will need the placement of a gastrostomy tube to help in supplementing caloric input and avoiding frequent chest infections in those patients with silent aspiration. Cardiac monitoring in the first two decades of life should focus on assessing silent cardiomyopathies or conductions defects, that might require medical treatment or implantable devices. Epilepsy should be monitored for and usually can be controlled with first-line antiepileptic drugs. While potential therapeutic interventions are in development for the LAMA2-RDs, disease-modifying medications are not available at present. It remains essential, however, that clinical management is optimized for both decreasing morbidity and improving clinical trial readiness.

## Biomarkers and Clinical Trial Needs in LAMA2-RD

Research in the LAMA2-RDs has benefited from the availability of multiple mouse models which, to varying degrees, recapitulate the human clinical phenotype of skeletal muscle weakness, respiratory insufficiency, and neuropathy. In particular, there are five separate mouse models of laminin-211 deficiency: *dy/dy*, *dy*^2J^/*dy*^2J^ and *dy*^nmf417^/*dy*^nmf417^ (spontaneous mutants); and *dy*^W^/*dy*^W^ and *dy*^3k^/*dy*^3k^ (generated mutants). The recognition of the involvement of apoptotic pathways as a potential disease mechanism in the LAMA2-RDs was demonstrated by studies performed in the *dy*^W^/*dy*^W^ mouse model in which the transgenic overexpression of *Bcl-2* (an apoptosis inhibitor) or inactivation of proapoptotic *Bax* resulted in prolonged survival (Girgenrath et al., 2004; Dominov et al., 2005). Preclinical studies were performed in the *dy*^W^/*dy*^W^ and *dy*^2J^/*dy*^2J^ mouse models of an antiapoptotic compound [N-(dibenz(b,f)oxepin-10-ylmethyl)-N-methyl-N-prop-2-ynylamine maleate] also known as TCH346 and omigapil, which binds to GAPDH thus inhibiting the Siah1-mediated nuclear translocation of GAPDH and the subsequent activation of the apoptotic pathway (Hara et al., 2005). Studies in the *dy*^W^/*dy*^W^ mouse demonstrated inhibition of GAPDH-Siah1-mediated apoptosis in muscle and improved locomotor activity, and studies in the *dy*^2J^/*dy*^2J^ mouse demonstrated decreased fibrosis in the skeletal muscles and the diaphragm muscle along with the improved respiratory rate. These findings formed the basis of the development of a phase 1 pharmacokinetic and safety study of omigapil in the LAMA2-RDs, the first clinical trial for this patient population (Erb et al., 2009; Yu et al., 2013). This study was an open-label, sequential group, ascending oral dose, cohort study with patients with either LAMA2-RD or COL6-related dystrophy (COL6-RD) who were stratified by disease type and weight and randomly assigned to one of three pre-specified dose cohorts. Omigapil was administered at a dose of 0.02–0.08 mg/kg/day for 12 weeks duration, and slightly greater than dose-proportional increases in systemic exposure to omigapil were seen. Overall, omigapil was found to be safe and well-tolerated, and the dose selected to achieve exposure within the pre-established target of the AUC0-24h range was found to be 0.06 mg/kg/day (Clinicaltrials.gov Identifier NCT01805024). Given the short duration of the study, no consistent changes were seen in disease-relevant clinical assessments; however, the study enabled the collection of pulmonary and motor outcome measures and provided an opportunity for the collection and investigation of exploratory outcome measures, including muscle ultrasound, muscle MRI and serum and urine biomarker collection.

Data for additional therapeutic approaches for LAMA2-RDs have emerged including from preclinical studies of transgenic expression of mini-agrin (mag) together with laminin-alpha1 LN-domain nidogen-1(alphaLNNd) in the *dy*^W^/*dy*^W^ mouse model which have demonstrated the restoration of basement membrane stability and the survival of this mouse model to over 2 years (5 times the typical survival). Given that the cDNA for mag and alphaLNNd can each fit into AAV vectors, and AAV vectors are utilized for transgene delivery for gene therapy studies for other muscle diseases presently in progress (NCT03362502, NCT03375164, NCT03652259, NCT03199469), this approach carries high promise for the potential of translation into clinical trials (Reinhard et al., 2017). Work demonstrating that transgenic overexpression of laminin α1 in the *dy*^3k^/*dy*^3k^ mouse model of laminin α1 deficiency improved muscle histological appearance, health, and longevity of the mice (Gawlik et al., 2004; Gawlik and Durbeej, 2010) and that transgenic overexpression of laminin α1 in the *dy*^2J^/*dy*^2J^ mouse model decreased disease severity (Gawlik et al., 2018) led to the subsequent development of a therapeutic approach of using the compensatory upregulation of endogenous Lama1 via AAV9 delivered, catalytically inactivated Cas9 (dCas9) linked VP64 transactivators and multiple guide RNAs in the *dy*^2J^/*dy*^2J^ mouse model (Kemaladewi et al., 2019). Another therapeutic approach developed based on findings from the transgenic overexpression of laminin α1 (Gawlik et al., 2004; Gawlik and Durbeej, 2010; Gawlik et al., 2018) is a protein substitution therapy approach, which has been studied in the *dy*^W^/*dy*^W^mouse model via the systemic delivery of the embryonically present laminin-111 protein, which has demonstrated stabilization of the basement membrane (Rooney et al., 2012). Protein therapy with laminin-111 was also studied in a *lama2*^−/−^ zebrafish model of LAMA2-RD and was reported to prevent contraction-induced damage to myofibers and promote reattachment of myofibers to the extracellular matrix (Hall et al., 2019).

### Natural History Studies

Collectively, these promising therapeutic avenues currently in development have galvanized efforts to better define the natural history of LAMA2-RDs and to identify viable clinical outcome measures to improve clinical trial readiness for individuals with LAMA2-RD. Identifying outcome measures that correlate with muscle function and are sensitive to change over time is an essential component of clinical trial readiness. One such effort to identify disease-specific outcome measures was a prospective study performed at the National Institutes of Health which measured longitudinal changes in LAMA2-RD patients (*n* = 24) and COL6-RD patients (*n* = 23) ages 4–22 years using the Motor Function Measure 32 (MFM32), myometry (of knee flexors and extensors and elbow flexors and extensors) and goniometry (of knee and elbow extension), pulmonary function tests and quality of life measures with five annual assessments. This study found that the MFM32 was sensitive to change in individuals with LAMA2-RD or COL6-RD in ambulatory and non-ambulatory children and adults. In particular, for non-ambulatory patients with LAMA2-RD, the rate of decline in total MFM32 score was −2.16 points/year (*p* < 0.01). In terms of longitudinal myometry measurements in LAMA2-RD patients, knee flexion strength in non-ambulatory patients declined by 2.47% per year (*p* < 0.01). Longitudinal goniometry measurements were statistically significant in non-ambulatory LAMA2-RD patients in left elbow extension (−4.11° per year; *p* < 0.01). The annual rate of change in forced vital capacity (FVC) was not found to be significant in LAMA2-RD patients in this longitudinal study; however, one potential reason could be that the non-ambulatory patients with LAMA2-RD in this study may have already reached a nadir in their respiratory insufficiency, resulting in a lack of further decline during this study which took place over 4 years (Jain et al., 2019). It is notable that in a separate, retrospective study of pulmonary function in 65 patients with LAMA2-RD, the annual rate of decline of FVC in non-ambulatory LAMA2-RD patients was found to be 1.73% per year (*p* < 0.01; Collins et al, personal observation). Currently, efforts are underway to perform a prospective natural history study focused on LAMA2-RD patients less than 5 years of age. This study is being coordinated among several specialist neuromuscular centers internationally to maximize the cohort size and thus increase the quantity of data collected. Given the paucity of early natural history data in LAMA2-RD and the goal of promising preclinical research efforts to translate into treatments aimed at treating patients with LAMA2-RD from a young age, this early natural history study is timely.

### Disease Biomarkers

Adequate, disease-relevant biomarkers, which could be capable of reflecting potential therapeutic changes in individuals with LAMA2-RD are also imminently needed. Such biomarkers would ideally provide a measure of disease and target engagement in patients participating in clinical trials without necessitating sequential muscle biopsies (Szigyarto and Spitali, 2018). In theory, developing comprehensive omics platforms may provide the best chance of identifying disease-relevant biomarkers. Investigations into proteomic evaluations for monitoring disease progression and predicting clinical course have been performed using samples from individuals with Duchenne muscular dystrophy (DMD) and Becker muscular dystrophy (BMD). These proteomic evaluations were used as a non-invasive way of monitoring disease progression and predicting future clinical courses (Spitali et al., 2018).

In a study analyzing the proteomic signature of the vastus lateralis muscle in four patients with genetically confirmed LAMA2-RD, 86 proteins were identified of which 35 were increased and 51 decreased [using liquid chromatography with tandem mass spectrometry (LC-MS/MS)] (Kölbel et al., 2019). Using the following bioinformatics programs: the Database for Annotation, Visualization, and Integrated Discovery (DAVID^2^), Kyoto Encyclopedia of Genes and Genomes (KEGG^3^), Reactome^4^ and Proteomap^5^ pathway analyses were performed. This data was then analyzed along with proteomic data from diaphragm and gastrocnemius muscles of the *dy*^3k^/*dy*^3k^ mouse model and identified nine common proteins that appear to be vulnerable, namely decreased proteins related to mitochondrial function in both (de Oliveira et al., 2014). Overall, this study offered preliminary data, for which more comprehensive studies are needed to more definitively identify tissue biomarkers of LAMA2-RD, including at particular stages of disease (Kölbel et al., 2019).

To be able to identify relevant biomarkers from thousands of candidates, networks and consortia with a data-sharing agreement and a common goal in mind need to be established. One such effort has resulted in the creation of Muscle Gene Sets (MGS^6^), which is also accessible via Enrichr, MsigDB/GSEA, and WebGestalt has provided a tool for functional genomics in neuromuscular conditions (Malatras et al., 2019). While not yet extended to proteomic data, this tool offers the ability to study the behavior of genes lists across more than 1,100 comparisons of muscle conditions. Extending such network and consortium efforts to tissue biobanks would provide the opportunity for using platform-type approaches for analyzing proteomic data, thus leveraging the collective efforts of various muscle research groups resulting in increased protein-protein interaction/interactome level data.

Beyond the prospect of identifying specific serum and protein biomarkers for LAMA2-RDs remain the prospect of muscle imaging modalities to serve as disease biomarkers, as well. Efforts towards performing quantitative magnetic resonance imaging (qMRI) to quantify fat replacement in muscular dystrophies include methods of chemical shift imaging (Dixon or IDEAL) or spectroscopy (Burakiewicz et al., 2017). In particular, qMRI of fat replacement has been found to have higher sensitivity than clinical assessments for capturing the progression of neuromuscular disease, including in DMD using two-point Dixon (Bonati et al., 2015) and transverse relaxation time constant (MRI-T2; Willcocks et al., 2016), LGMD type 2I using two-point and three-point Dixon (Willis et al., 2013), oculopharyngeal muscular dystrophy using two-point Dixon (Fischmann et al., 2012), and inclusion body myositis and Charcot-Marie-Tooth disease 1A using three-point Dixon (Morrow et al., 2016). While not evaluated in comparison to motor outcome measure assessments, muscle MRI has been performed in LAMA2-RD patients with preliminary quantitative MRI-T2 and MRS fat fraction evaluations performed (Walter et al, personal observation). If the challenges which joint contractures and dependency on non-invasive ventilation pose to comfortable positioning and stable ventilation during the MRI could be overcome, further use of qMRI in the LAMA2-related population could be evaluated and, such as in other muscular dystrophies, may prove to be highly sensitive to disease progression.

The identification of biomarkers sensitive and specific enough for measuring clinical benefit could enable a smoother road for demonstrating potential efficacy or lack of efficacy of particular interventions, thus improving the efficiency of the journey of translational research along the so-called “bench-to-bedside” pipeline for bringing promising therapeutics to patients. It is important to note that in LAMA2-RD caused by biallelic loss-of-function mutations and resulting in a “null” status for the LAMA2 protein/complete merosin deficiency, the onset of the disease is before birth. Thus, clinical and pathological evidence of the disease is already established at birth, at which time severe muscle weakness and muscle biopsy evidence of degeneration and inflammation are seen (Pegoraro et al., 1996). It is therefore essential that therapeutic efforts for the LAMA2-RDs be focused on targeting patients at a very early age and be robust enough in their potential therapeutic effects to demonstrate target engagement and clinical improvements in patients with established disease symptoms at birth. Clinical trial readiness for individuals with LAMA2-RD will depend on combining biobank efforts in a joint quest of identifying disease-relevant biomarkers which are capable of capturing potential changes due to therapeutic interventions. The promising therapeutic approaches in preclinical development for LAMA2-RD all share the overall goal of resulting in meaningful clinical improvements for individuals affected by this CMD subtype. To this end, clinical trial endpoints- as measured via outcome measures- need to be capable of capturing biological and physical improvements which are meaningful to patients as well as recognized as significant from the perspective of regulatory agencies, who will ultimately determine which therapeutics are approved and thus made available for use by the entire patient population.

## Ethics Statement

Written informed consent was obtained from the parent of the patient for the publication of the clinical image included in this article.

## Author Contributions

AS and AF equally contributed to the conception and design of the article, acquisition of data, drafting and critical revision of the final manuscript. AZ contributed to the literature review and drafting of the manuscript. CB and FM contributed to the conception and design of the article, critical revision of the manuscript, review, and approval of the final version.

## Conflict of Interest

The authors declare that the research was conducted in the absence of any commercial or financial relationships that could be construed as a potential conflict of interest.

## Footnotes

^1^https://databases.lovd.nl/shared/genes/LAMA2

^2^www.david.ncifcrf.gov

^3^www.genome.jp/kegg/pathway.html

^4^www.reactome.org

^5^www.proteomaps.net

^6^http://www.sys-myo.com/muscle_gene_sets/
